# Bilateral Choroid Plexus Papillomas Diagnosed by Prenatal Ultrasound and MRI

**DOI:** 10.7759/cureus.13737

**Published:** 2021-03-06

**Authors:** Yi Li, Shilpa Chetty, Vickie A Feldstein, Orit A Glenn

**Affiliations:** 1 Radiology and Biomedical Imaging, University of California San Francisco, San Francisco, USA; 2 Obstetrics and Gynecology, University of California San Francisco, San Francisco, USA

**Keywords:** choroid plexus papilloma, choroid plexus, fetal mri, doppler ultrasound, choroid plexus hyperplasia

## Abstract

We present a rare prenatal diagnosis of bilateral choroid plexus papillomas by obstetrical ultrasound and fetal MRI at 20 weeks 6 days gestation. The fetus demonstrated bilateral enlarged, echogenic choroid plexus with increased Doppler flow suggestive of vascularized choroid tissue. Same-day fetal MRI demonstrated that the choroid plexus appeared enlarged bilaterally without definite hemorrhage. The combined features on ultrasound and MRI suggested bilateral choroid plexus papillomas with increased cerebrospinal fluid production, leading to ventriculomegaly and enlarged extra-axial spaces. The diagnosis was confirmed by postnatal pathology, which demonstrated WHO grade II atypical choroid plexus papillomas.

## Introduction

Choroid plexus tumors are neuroectodermal tumors of the choroid plexus epithelium, most commonly found in the atria of the lateral ventricles. Choroid plexus papillomas represent the lowest grade of choroid plexus tumor (WHO grade I). Higher grade tumors in this category include atypical choroid plexus tumors (WHO grade II) and choroid plexus carcinoma (WHO grade III) [1]. These tumors are most common in the pediatric population and quite rare in adults. Approximately 10% of all brain tumors in infants and 5% of perinatal brain tumors are of choroid plexus etiology [2]. In this report, we present a case of bilateral choroid plexus papillomas diagnosed in utero by fetal ultrasound and fetal MRI and confirmed by postnatal pathology.

## Case presentation

**Figure 1:**
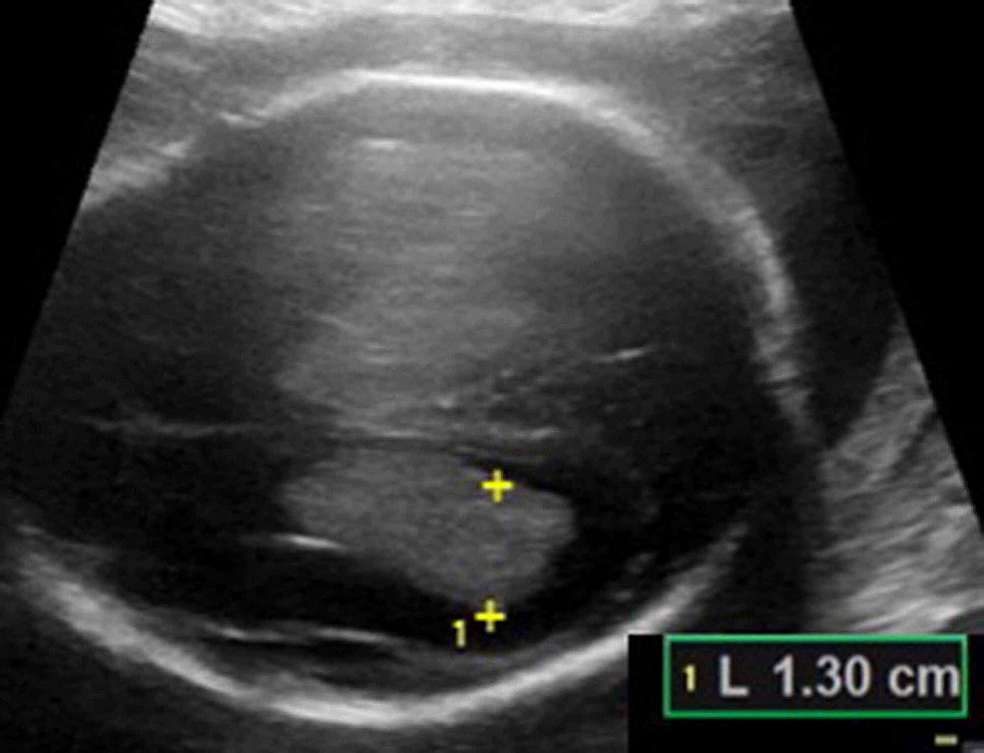
Transverse sonographic image of the fetal brain at 20 weeks 6 days, obtained by transabdominal ultrasound Enlarged bilateral choroid plexus, with associated ventriculomegaly is seen; left lateral cerebral ventricle measures 13 mm in atrial diameter.

A 29-year-old gravida 4, para 3 female with a past medical history notable for type 2 diabetes and elevated HgbA1c at 7.5% was first noted to have a fetal abnormality by nuchal translucency ultrasound (US) at an outside institution, with nuchal translucency measuring 3.8 mm. She underwent genetic counseling but declined further invasive testing. Cell-free DNA screening was performed and results were low risk for Down syndrome, trisomy 13 or 18. Given these abnormalities, however, she underwent further fetal ultrasound at 16 weeks and was noted to have ventriculomegaly with a suspected posterior fossa mass. She presented to our institution at 20 weeks 6 days gestation, and fetal US and brain MRI were performed. Obstetric ultrasound demonstrated symmetric bilateral enlargement of echogenic choroid plexus with associated ventriculomegaly and enlarged extra-axial fluid-filled spaces (Figure 1), with differential diagnosis including intraventricular hemorrhage, choroid plexus hyperplasia or choroid plexus papilloma. Head size was larger than expected for gestational age (GA). Biparietal diameter measured 6.7 cm (corresponding with 27 weeks 2 days GA) and head circumference measured 24.2 cm (corresponding with 26 weeks 1 day GA).

**Figure 2:**
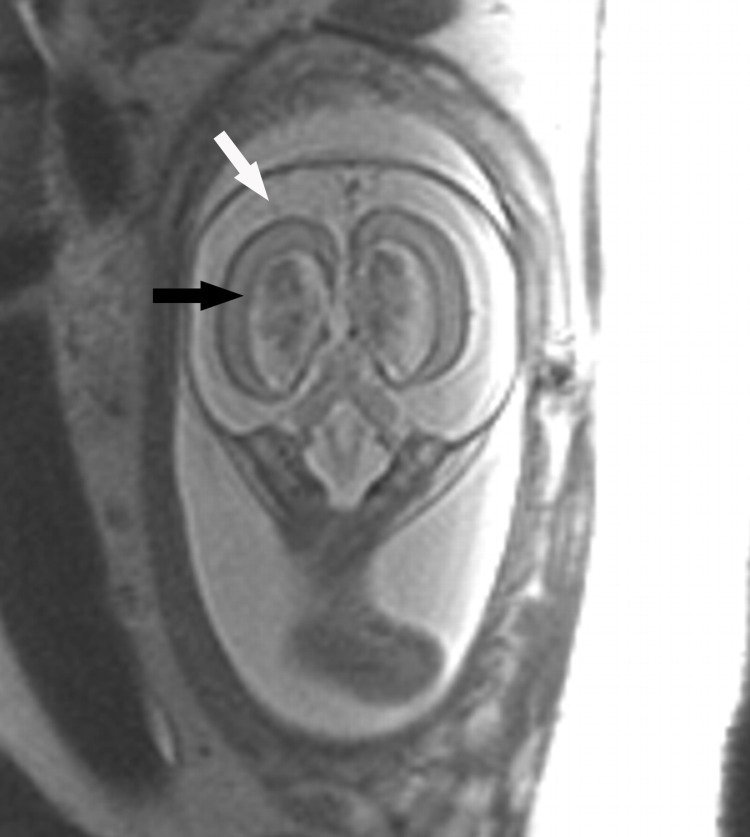
Coronal T2-weighted single-shot fast spin-echo of the fetal brain at 20 weeks and 6 days. Coronal T2-weighted single-shot fast spin-echo of the fetal brain at 20 weeks 6 days demonstrates enlargement of the lateral ventricles (black arrow), filled with prominent choroid plexus. Note also made of enlarged bilateral extra-axial spaces (white arrow). Parenchymal thinning was present, likely secondary to ventriculomegaly.

An MRI scan of the fetal brain performed on the same day demonstrated bilateral ventriculomegaly (Figure 2). The lateral ventricles measured up to 14 mm in atrial diameter, with normal morphology of the third and fourth ventricles. There was also marked enlargement of the supratentorial and infratentorial extra-axial subarachnoid spaces. The choroid plexus appeared enlarged bilaterally without definite hemorrhage on echo-planar imaging. Additionally, there was no susceptibility or T2 hypointensity outlining the ventricles or brainstem, suggesting that there was no prior intracranial hemorrhage.

Based on these MRI findings, targeted Doppler US was performed and demonstrated increased flow within the choroid plexus bilaterally. Thus, the US findings suggested enlarged vascularized choroid, rather than intraventricular hemorrhage. No other fetal morphologic abnormality was noted. Mild parenchymal thinning was present, likely secondary to ventriculomegaly. The findings on prenatal US and fetal MRI suggested bilateral choroid plexus papillomas or hyperplasia with increased cerebrospinal fluid (CSF) production leading to hydrocephalus and enlarged subarachnoid spaces. The differential diagnosis included possible sequelae of dural venous sinus atresia, leading to decreased CSF absorption and engorgement of the choroid plexuses, and a follow-up study was recommended.

**Figure 3:**
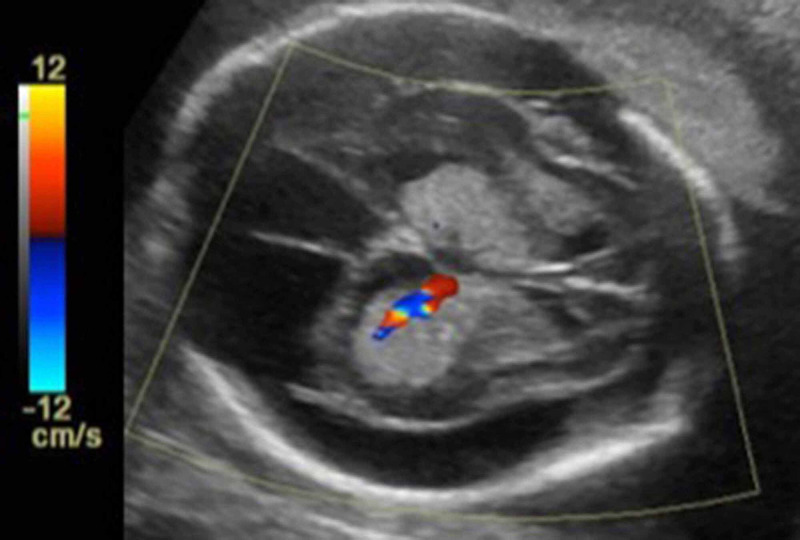
Follow-up color Doppler ultrasound at 22 weeks 6 days, obtained by transabdominal ultrasound, shows progressive ventriculomegaly with notably enlarged extra-axial spaces There is increased vascularity of the echogenic choroid plexus. This finding helps corroborate that the enlargement is due to choroid plexus papillomas, rather than intraventricular hemorrhage.

Follow-up sonogram at 22 weeks 6 days gestation demonstrated marked macrocephaly (head circumference 30.4 cm corresponding with 33 weeks 1 day GA) with further increase in the size of bilateral choroid plexuses and of extra-axial CSF volume. Prominent flow was again shown by Doppler US within the enlarged choroid plexus (Figure 3), making bilateral choroid plexus papillomas or hyperplasia the most likely diagnoses. Choroid plexus papilloma was favored, due to the relative rarity of choroid plexus hyperplasia [3].

The fetal head size continued to enlarge. On obstetric sonogram at 31 weeks 3 days, the biparietal diameter measured 22.6 cm and head circumference measured 75.3 cm, both significantly beyond standard measurements of fetal head size.

**Figure 4:**
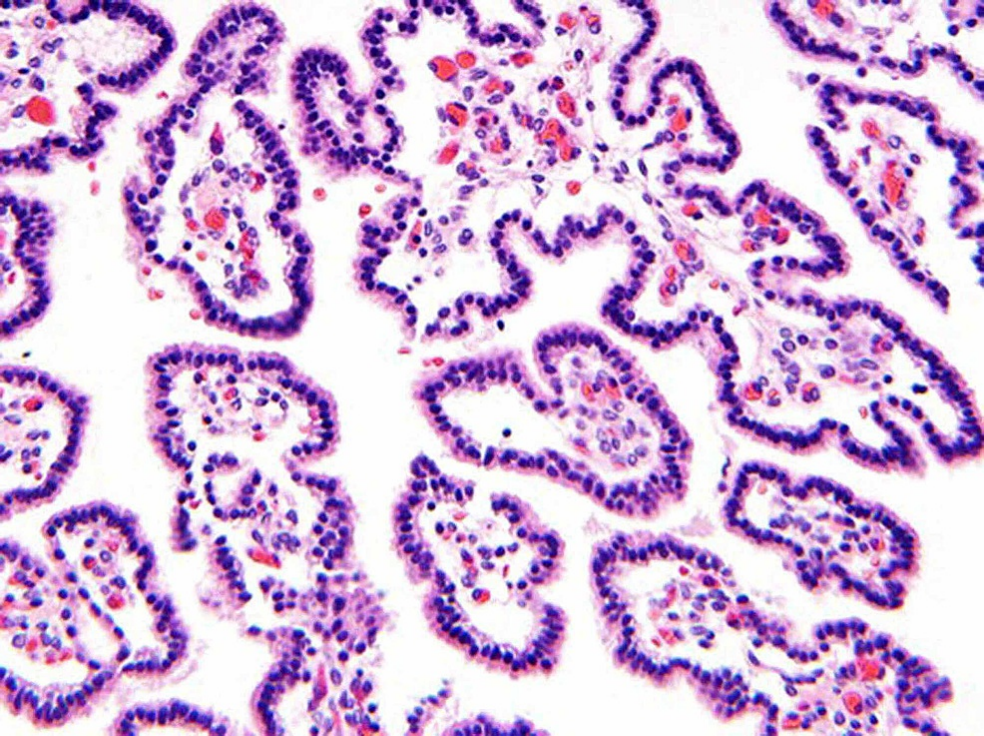
Hematoxylin and eosin (H&E)-stained section of the tumor revealing an atypical choroid plexus papilloma Courtesy: David A. Solomon, MD, PhD, Department of Pathology at University of California San Francisco, contributed the histology image and description. Fibrovascular papillary projections are lined by a single layer of cuboidal epithelium, which demonstrated immunopositivity for S-100, glial fibrillary acidic protein, and transthyretin (not shown). Elevated mitotic activity (up to 4 mitoses per 10 high-power fields) and Ki67 labeling index of approximately 15% warranted designation of this congenital choroid plexus papilloma as atypical (WHO grade II).

The patient presented in preterm labor at 31 weeks 6 days. Due to marked macrocephaly, fetal cephalocentesis was performed at the time of delivery. Under ultrasound guidance, a 22-gauge spinal needle was introduced through the uterine wall, through the fetal scalp and into the enlarged extra-axial CSF space of the fetal head. The spinal needle was connected to a vacuum bottle, with controlled drainage of 3.5 L of xanthochromic CSF. Post-procedural ultrasound demonstrated no evidence of intracranial hemorrhage. The fetus was subsequently delivered by cesarean section, but demised shortly after delivery, despite efforts at resuscitation. Postnatal pathology confirmed atypical choroid plexus papillomas with ependymal differentiation (WHO grade II; Figure 4).

## Discussion

Choroid plexus papillomas are among the most common brain tumors in children younger than two years of age [4]. Approximately 10% of all brain tumors in infants and 5% of perinatal brain tumors are of choroid plexus etiology [2]. A few case reports of congenital choroid plexus tumors diagnosed in utero exist in the literature [4-10]. In all cases, ultrasound demonstrated large, echogenic choroid plexus with associated ventriculomegaly. In most of these cases, the papillomas were unilateral, with only one prior bilateral case reported [7]. Additionally, in most cases, the diagnosis was suspected on the basis of prenatal ultrasound in the third trimester, without fetal MRI [5, 6,8-10]. In one case, however, fetal MRI was used in the third trimester to confirm the presence of a unilateral intraventricular mass without hemorrhage [4]. In another report, although sonographic diagnosis was suspected in the second trimester, only postmortem fetal MRI was performed to confirm the diagnosis, and in utero fetal MRI was not done [7].

In the case reported here, the diagnosis of bilateral choroid plexus papillomas was prospectively diagnosed in utero during the second trimester, using a combination of fetal ultrasound and fetal MRI findings. There was no convincing MR evidence of intraventricular or choroid plexus hemorrhage, and the Doppler vascularity of the enlarged choroid plexus made mass more likely than hemorrhage. Of bilateral choroid plexus masses, papilloma is more common than hyperplasia [3]. The prenatal diagnosis of choroid plexus hyperplasia is rare, with one prior report in the literature that demonstrated a benign course but no postnatal pathologic diagnosis [11]. Several childhood pathology-confirmed diagnoses of choroid plexus hyperplasia exist in the literature. In one recent report, the authors distinguish choroid plexus hyperplasia from papilloma based on moderate enhancement of the enlarged choroid plexus in hyperplasia, as opposed to avid enhancement typically seen in papillomas [12]. Contrast, however, is not administered for fetal MRI. In our case, the presumptive diagnosis of bilateral choroid plexus papilloma was made in utero based on the relatively higher prevalence of papilloma compared to hyperplasia.

Our case demonstrates similar imaging features compared to the prior reports, but differs from those in the literature due to the presence of bilateral papillomas. Additionally, we imaged the papillomas by fetal MRI in the second trimester, rather than in the third trimester or postnatally. The combination of imaging findings on simultaneously acquired fetal ultrasound and MRI helped narrow down the differential diagnosis and increased diagnostic confidence regarding this rare diagnosis. Follow-up obstetric ultrasound revealed expected progression and added diagnostic confidence as well. In utero diagnosis of this condition allowed for appropriate patient counseling, triage and management at the time of delivery.

Atypical choroid plexus papillomas tend to carry a worse prognosis than grade I choroid plexus papillomas, but a better prognosis than choroid plexus carcinoma. Previously published survival rates for perinatal atypical choroid plexus papilloma is 67%, which is intermediate between 81% for choroid plexus papilloma and 45% for choroid plexus carcinoma [2]. If patients survive the neonatal period, the treatment regimen for this tumor typically involves surgical resection, and when indicated and feasible, may also include adjuvant or neoadjuvant chemotherapy, and/or preoperative embolization, to reduce tumor size and vascularity [13,14]. Radiation is not typically used in the neonatal population due to long-term deleterious effects on developing tissue [2].

## Conclusions

In this report, we present the fetal ultrasound and MRI findings of a rare case of prenatally diagnosed bilateral choroid plexus papilloma. The fetus demonstrated bilateral enlarged, echogenic choroid plexus with increased Doppler flow suggestive of vascularized choroid tissue. Same-day fetal MRI demonstrated that the choroid plexus appeared enlarged bilaterally without definite hemorrhage. The constellation of findings on ultrasound and MRI suggested bilateral choroid plexus papillomas, with increased CSF production, leading to ventriculomegaly and enlarged extra-axial spaces. This diagnosis was confirmed by postnatal pathology. Fetal bilateral choroid plexus papilloma is a rare diagnosis, but should be kept in mind with this imaging appearance. In this case, prenatal diagnosis allowed for appropriate patient counseling, triage and management at the time of delivery.
